# Do face masks help? is not the question

**DOI:** 10.1073/pnas.2012241117

**Published:** 2020-10-06

**Authors:** Qin Xiang Ng, Michelle Lee Zhi Qing De Deyn, Wee Song Yeo

**Affiliations:** ^a^MOH Holdings Pte Ltd., Singapore 099253;; ^b^Department of Medicine, James Cook University Hospital, Middlesbrough TS4 3BW, United Kingdom;; ^c^Department of Pediatric Medicine, Mount Elizabeth Hospital, Singapore 228510

We read with great interest the study by Zhang et al., which analyzed the trend in the daily number of new confirmed cases and mitigation measures in three COVID-19 epicenters, namely Wuhan, China, Italy, and New York City (NYC) (1). The researchers concluded that social distancing is insufficient per se and wearing of face masks in public spaces is the most effective preventive measure. We appreciate the conclusions reached, but the analyses and conclusions are problematic for several reasons.

First, it is at best imprecise, and at worst grossly inaccurate, to attribute the slower increase in the numbers of daily new cases between NYC and the United States after 14 April to the implementation of wearing face masks in public. The authors did not consider lags in epidemic dynamics. We cannot conclude that facemasks alone or the extended lockdown alone (2) drove the reduction in the transmission rate. Their combined effect is what is being measured in the study. Due to the variable incubation period of COVID-19, which could take between 1 to 24 d (3), as well as testing and reporting delays, new cases and hospitalizations reflect individuals who were infected 1 to 2 wk prior. Furthermore, although Governor Andrew Cuomo urged and later signed an executive order mandating New Yorkers to cover their faces in public, when not practicing social distancing, the actual implementation and compliance on the ground has been widely questioned (4). There are no fines or penalties for noncompliant individuals, and enforcement is purely at the discretion of local jurisdictions. We know that “socially responsible” behaviors may not be intuitive and, as with health behavior change, take time. It is thought that it takes anywhere between 18 and 254 d to develop a new habit (5).

Second, Zhang et al. (1) oversimplify the day-to-day fluctuations and erroneously applied linear regression to the data between 17 April and 9 May in NYC and between 5 April and 9 May in the United States. Linear regression should be limited to variables with a linear relationship (6). In the case of COVID-19, modeling the disease rate as the virus spreads in the community is complex. The researchers used best-fit lines to support a decreasing rate in the daily new infections; we would advise caution in interpreting these linear regressions. Nonlinear regression analysis, or a more explicitly epidemiological model such as EpiEstim (7), would be more appropriate.

It is too premature to discount the preventive benefits of social distancing in favor of face masks. We do certainly hope that properly worn face masks help stem the spread of the coronavirus, and there is growing evidence to support the recommendation to wear masks in public spaces (8). However, ensuring public compliance is in itself a difficult task. In Singapore, stringent laws were put in place to enforce wearing face masks in public and work spaces; first-time offenders would be fined while egregious cases could be prosecuted in court (9).

## References

[1] R. Zhang, Y. Li, A. L. Zhang, Y. Wang, M. J. Molina, Identifying airborne transmission as the dominant route for the spread of COVID-19. Proc. Natl. Acad. Sci. U.S.A. 117, 14857–14863 (2020). 32527856 10.1073/pnas.2009637117 PMC7334447

[2] CBS News, “Lockdown extended for most of coronavirus-battered New York” (video recording, 2020). https://www.cbsnews.com/news/new-york-stay-at-home-extended-coronavirus-lockdown. Accessed 12 June 2020.

[3] S. A. Lauer., The incubation period of coronavirus disease 2019 (COVID-19) from publicly reported confirmed cases: Estimation and application. Ann. Intern. Med. 172, 577–582 (2020). 32150748 10.7326/M20-0504 PMC7081172

[4] N. MacFarquhar, Who’s enforcing mask rules? Often retail workers, and they’re getting hurt. *NY Times*, 15 May 2020. https://www.nytimes.com/2020/05/15/us/coronavirus-masks-violence.html. Accessed 12 June 2020.

[5] P. Lally, C. H. Van Jaarsveld, H. W. Potts, J. Wardle, How are habits formed: Modelling habit formation in the real world. Eur. J. Soc. Psychol. 40, 998–1009 (2010).

[6] R. J. Casson, L. D. Farmer, Understanding and checking the assumptions of linear regression: A primer for medical researchers. Clin. Exp. Ophthalmol. 42, 590–596 (2014). 24801277 10.1111/ceo.12358

[7] R. N. Thompson., Improved inference of time-varying reproduction numbers during infectious disease outbreaks. Epidemics 29, 100356 (2019). 31624039 10.1016/j.epidem.2019.100356 PMC7105007

[8] W. Lyu, G. L. Wehby, Community use of face masks and COVID-19: Evidence from a natural experiment of state mandates in the US. Health Affairs 39, 1419–1425 (2020). 32543923 10.1377/hlthaff.2020.00818

[9] M. L. Z. Q. De Deyn, Q. X. Ng, W. Loke, W. S. Yeo, A tale of two cities: A comparison of Hong Kong and Singapore’s early strategies for the Coronavirus Disease 2019 (COVID-19). J. Infect. 81, e51–e52 (2020). 10.1016/j.jinf.2020.06.058 PMC7316449 32593657

